# Voice biomarkers in middle and later adulthood as predictors of cognitive changes

**DOI:** 10.3389/fpsyg.2024.1422376

**Published:** 2024-10-18

**Authors:** Elizabeth Mahon, Margie E. Lachman

**Affiliations:** Psychology Department of Psychology, Brandeis University, Waltham, MA, United States

**Keywords:** middle age, cognitive performance, prosody voice, aging biomarkers, cognitive change

## Abstract

**Background:**

Prosody voice measures, especially jitter and shimmer, have been associated with cognitive impairment and hold potential as early indicators of risk for cognitive decline. Prior research suggests that voice measures assessed concurrently with longitudinal cognitive outcomes are associated with 10-year cognitive declines in middle-age and older adults from Midlife in the U.S. (MIDUS) study.

**Results:**

Using a subsample from the same study, we expanded previous research to examine voice measures that were (1) collected 8 years before cognitive outcomes, (2) derived from narrative speech in logical memory tests instead of word list recall tests, and (3) independent of the cognitive outcomes. Multilevel analyses controlled for covariates of age, sex, education, neurological conditions, depressive symptoms, and chronic conditions. The results indicated that higher jitter and lower shimmer predicted greater 10-year declines in episodic memory and working memory.

**Conclusion:**

These findings extend previous research by highlighting prosody voice measures assessed 8 years earlier as predictors of subsequent cognitive declines over a decade.

## 1 Introduction

As the aging population grows, identifying reliable indicators of cognitive changes across various life stages becomes increasingly important (Livingston et al., 2020). During the preclinical phase, subtle cognitive declines may resemble normal aging and usually do not disrupt daily activities. However, as cognitive impairment progresses, these declines become more pronounced and begin to significantly impact everyday life (Bryan and Maxim, 2006; Gerstner et al., 2007). Cognitive changes can occur 3–7 years before clinical symptoms warrant a diagnosis of cognitive impairment (Bateman et al., 2012). This issue is particularly critical, as disease pathology can develop long before cognitive impairments become evident (Livingston et al., 2020). Abnormal accumulation of amyloid plaques can begin 20–30 years before the clinical symptoms appear (Bateman et al., 2012; Sperling et al., 2011). Given that interventions are most effective when administered at an early stage an important objective in aging and dementia research is the identification of early warning signs. While various factors have been associated with the likelihood of cognitive decline and impairment, there is a continuing need to identify cost-effective and reliable biomarkers capable of detecting early indicators of cognitive risks. This study explores voice prosody as a potential biomarker that may be linked to the risk of cognitive decline.

Measures of voice have shown high sensitivity in distinguishing between normal cognition and stages of cognitive impairment (Beltrami et al., 2018; Kato et al., 2018; Themistocleous et al., 2020; Thomas et al., 2020; Toth et al., 2017; Xue and Deliyski, 2001). Among these metrics, prosody voice measures are particularly promising, as they can differentiate between individuals with normal cognitive function and others with different stages of impairment (Konig et al., 2018, 2019; Mirheidari et al., 2019; Testa et al., 2001). Key prosody measures such as pitch, pulse, voice breaks, jitter, shimmer, and amplitude have shown potential (Konig et al., 2018, 2019; Martínez-Sánchez et al., 2012; Meilán et al., 2014; Mirheidari et al., 2019; Nasreen et al., 2021; Nishikawa et al., 2022; Testa et al., 2001). These measures could complement existing diagnostic tools like MRI scans and blood tests due to their lower cost, less invasive, and ease of monitoring cognitive health over time (John et al., 2012; Karlamangla et al., 2005; Lu et al., 2015; Österberg et al., 2012; Paterson et al., 2018; Quadri et al., 2004; van Himbergen et al., 2012; Wolf et al., 2002).

Our recent research, which analyzed data from middle-aged and older adults, found that lower pulse and higher jitter were associated with greater cognitive decline over 10 years when assessed concurrently with cognitive outcomes (Mahon and Lachman, 2022). This finding suggests that voice biomarkers, which are already associated with cognitive impairment diagnoses, may also be effective in identifying individuals at higher risk for developing impairment. Therefore, voice alterations may help differentiate between cognitive declines related to normal aging and steeper declines progressing to severe impairment. However, additional research is needed to determine the effectiveness of these metrics as indicators of early preclinical stages. Ultimately, leveraging voice biomarkers already connected to cognitive impairments could enhance our ability to identify individuals at increased risk of cognitive decline and progression to impairment later in life (Bondi et al., 1999).

This study investigates prosody voice as a potential biomarker for cognitive decline. Our previous research assessed prosody measures concurrently with 10-year cognitive outcomes, revealing associations between lower pulse and higher jitter and cognitive decline (Mahon and Lachman, 2022). The current study expands on this work in 3 ways: (1) Instead of assessing voice concurrently with cognitive outcomes, we measured voice 8 years before to cognitive outcomes to examine predictive associations; (2) Unlike our previous work, voice data was derived from recorded cognitive interviews that were not included as longitudinal cognitive measures in our study, addressing potential concerns that voice features may reflect level of test performance; and (3) We analyzed voice data from narrative speech during recall of a story rather than recall of a word list. We hypothesized that lower pulse and higher jitter, measured 8 years prior to cognitive outcomes, would be associated with greater cognitive decline across 10 years, after adjusting for demographic and health variables. Additionally, based on past research, we expect that higher pitch, higher voice breaks, higher shimmer, and lower amplitude would be associated with greater cognitive decline.

## 2 Methods

### 2.1 Participants

Participants in this study were a subset of the Midlife in the United States Study (MIDUS), who were included if they had cognitive data from both MIDUS 2 (M2) and MIDUS 3 (M3), with a mean lag time of 96–120 months (M = 109.79 months ± 5.32). Our inclusion criteria also required participants to have audio recordings from the Boston Longitudinal Study (BOLOS) subsample (*N* = 79), which were collected 3–45 months after M2 (M = 10.98 months ± 8.01) and 65–111 months before M3 (M = 98.62 months ± 9.27). Additional information about the samples used in this study can be found in prior publications (Radler and Ryff, 2010; Agrigoroaei and Lachman, 2011; Hughes et al., 2018). At the time of M2, our analysis sample ranged in age from 34 to 82 years (M = 57.94 ± 11.75), were 51.9% women, and had an average education of 15.50 years.

### 2.2 Cognitive measures

The cognitive measures from M2 (2004–2005) and M3 (2013–2014) were from the Brief Test of Adult Cognition by Telephone (BTACT; Tun and Lachman, 2006; Lachman and Tun, 2008; Lachman et al., 2014). The BTACT evaluated a range of cognitive functions: episodic memory was measured with the Word List Immediate and Delayed tests; inductive reasoning was measured with the Number Series test; category verbal fluency was measured with the Category Fluency test; working memory span was measured with the Backward Digit Span test; processing speed was measured with the 30 Second and Counting Task test; and attention switching reaction time and inhibitory control was measured with latencies from the Stop and Go Switch Task test. Additional information can be found in prior publications (Lachman and Tun, 2008; Hughes et al., 2018).

### 2.3 Voice measures

Six prosody voice measures were averaged across 2 Logical Memory tests (narrative speech from immediate and delayed story recall) that were not used as cognitive measures in this study. Pitch was measured by mean number of vocal cord vibrations per second; pulse was measured by number of glottal pulses of air; number of voice breaks was measured by total time of breaks/by total time of voice; jitter was measured by frequency instability; shimmer was measured by amplitude instability; and amplitude was measured by average noise-to-harmonics ratio. These voice measures were extracted from 2 tests in the BOLOS subsample interview (2004–2005) which had the longest uninterrupted segments of participant voice from Logical Memory Immediate and Delayed tests (Weschsler, 1981), with a total of 79 recordings. Each research recording was randomly assigned to 2 separate research assistants, who identified the voice segment and voice analyzed it with Praat voice analysis software (Boersma and Weeink, 2019); the interrater reliability was 98.21%, with any discrepancies resolved by the author through reanalysis of the voice segments. Only recordings that were of high quality and complete for cognitive assessments were included (*n* = 79). The correlations of the Praat measures across the 2 Logical Memory tests were as follows: pitch, *r*(79) = 0.781, (*p* = <0.001), pulse, *r*(79) = 0.406, (*p* = <0.001), voice breaks, *r*(79) = 0.204, (*p* = 0.088), jitter, *r*(79) = 0.748, (*p* = <0.001), shimmer, *r*(79) = 0.709, (*p* = <0.001), and amplitude, *r*(79) = 0.717, (*p* = <0.001). Voice measures were averaged across the 2 tests to create composites.

### 2.4 Covariates

Consistent with our previous study, we included covariates from M3: self-reported age (continuous), sex (male = 0, female = 1), education (in years), depressive symptoms (0 = no, 1 = yes), the number of neurological conditions (a count of “yes” responses to 4 health conditions: stroke, serious head injury, Parkinson’s disease, or other neurological disorder), and the number of chronic conditions (a count of “yes” responses to 22 health conditions). Additional information on these covariates can be found in our prior publication (Mahon and Lachman, 2022).

### 2.5 Data analyses

Descriptive statistics were conducted in SPSS 28.0 (IBM Corp, 2023). Longitudinal multi-level modeling (MLM; Bolger and Laurenceau, 2013) and the lme4 package (Bates et al., 2011) in R (R Core Team, 2021) was employed to examine the relationships between voice measures and 10-year cognitive changes. Time was coded linearly as 0 = M2 cognition and 1 = M3 cognition. The analysis controlled for age, sex, education, neurological conditions, depressive symptoms, and chronic conditions. Cognitive measures from M2 and M3 were standardized using the means and standard deviations of M2 cognitive scores. To maintain consistency in directional interpretation across cognitive tests, the SGST Latency variable was multiplied by (−1), so that higher scores indicated better (i.e., faster) performance.

Each of the 7 cognitive tests were analyzed using the following multi-level model:


(1) $$\begin{array}{l} Cognitive\_score_{ti}=\beta_{0i}+\beta_{01}\left(pitch_{i}\right)+\beta_{02}\left(pulse_{i}\right)+\\ \beta_{03}\left(voice\_breaks_{i}\right)+\beta_{04}\left(jitter_{i}\right)+\\ \beta_{05}\left(shimmer_{i}\right)+\beta_{06}\left(amplitude_{i}\right)+\\ \beta_{07}\left(time_{ti}\right)+\beta_{08}\left(pitch_{i}\right)\left(time_{ti}\right)+\\ \beta_{09}\left(pulse_{i}\right)\left(time_{ti}\right)+\\ \beta_{10}\left(voicebreaks_{i}\right)\left(time_{ti}\right)+\\ \beta_{11}\left(jitter_{i}\right)\left(time_{ti}\right)+\\ \beta_{12}\left(shimmer_{i}\right)\left(time_{ti}\right)+\\ \beta_{13}\left(amplitude_{i}\right)\left(time_{ti}\right)+\beta_{14}\left(age_{i}\right)+\\ \beta_{15}\left(sex_{i}\right)+\beta_{16}\left(education_{i}\right)+\\ \beta_{17}\left(neurological\_conditions_{i}\right)+\\ \beta_{18}\left(depressive\_symptoms_{i}\right)+\\ \beta_{19}\left(chronic\_conditions_{i}\right)+e_{ti}+e_{0i} \end{array}$$

Equation 1 represents the simple linear growth model incorporating the interaction effect of time based on the 2 waves of cognitive test scores for the i-th participant: time (t) represents the 2 waves (0 = M2 cognition and 1 = M3 cognition). β_0i_ is the average cognitive test score at M2 cognition when each covariate equals zero. Power analysis (Moineddin et al., 2007) indicated that the observed effect size for all 12 predictor variables of voice measures and covariates, measured by Cohen’s d, was d = 0.57, indicating a medium effect size. Using a multi-level modeling approach with 6 primary predictors, we achieved the desired power level of 80% and a significance level of *p* = 0.05. Based on these research findings, a sample size of at least 31 participants was determined to be necessary.

## 3 Results

Descriptive statistics are presented in Table 1, and multi-level model results are displayed in Table 2. As predicted, higher jitter was significantly associated with greater declines in Word List Immediate (*p* = 0.018), Word List Delayed (*p* = 0.015), and Backward Digit Span (*p* = 0.022). Contrary to expectations, lower shimmer was significantly associated with greater declines in Word List Immediate (*p* = 0.017), Word List Delayed (*p* = 0.023), and Backward Digit Span (*p* = 0.008). Additional analyses that included the lag time between M2 and BOLOS time points, as well as the lag time between BOLOS and M3 time points, produced consistent results.

**Table 1:** Descriptive statistics for our analysis sample.

	*N*	Minimum	Maximum	Mean	Std. Deviation
Pitch	79	86.94	264.35	155.71	37
Pulse	79	292.50	2,724	1251.43	582.25
Voice breaks	79	17	128.50	56.01	23.04
Jitter	79	1.52	8.85	3.28	1.11
Shimmer	79	10.30	23.13	17.46	2.96
Amplitude	79	0.13	0.55	0.32	0.09
M2 age	79	34	82	57.94	11.75
Sex (51.90% women)	79				
Education (years)	79	8	20	15.50	2.82
M3 neurological conditions	79	0	2	0.22	0.45
M3 depressive symptoms (11.4% yes)	79				
M3 chronic conditions	78	0	8	2.53	2.13
M2 word list immediate	78	3	11	7.04	1.80
M2 word list delayed	76	0	9	4.37	2.24
M2 category fluency	78	7	36	20.79	6.31
M2 backward digit span	78	3	8	5.62	1.39
M2 number series	78	0	5	3.03	1.44
M2 30 second and counting task	78	0	78	39.47	11.46
M2 stop and go switch task	74	−2	−0.72	−1.037	0.20
M3 word list immediate	79	2	12	6.77	2.23
M3 word list delayed	75	0	9	4.40	2.73
M3 category fluency	78	0	33	19.59	6.35
M3 backward digit span	79	0	8	4.90	1.68
M3 number series	75	0	5	2.68	1.49
M3 30 second and counting task	77	16	70	37.30	10.01
M3 stop and go switch task	73	−3.59	−0.70	−1.302	0.42

**Table 2:** Multilevel model results for prosody voice variables.

Cognitive Test	B	SE	*p*	CI_95_
Word list immediate				
Time × jitter^*^	−0.755	0.312	0.018	−1.376; −0.134
Time × shimmer^*^	0.325	0.133	0.017	0.059; 0.591
Word list delayed				
Time × jitter^*^	−0.915	0.366	0.015	−1.643; −0.183
Time × shimmer^*^	0.366	0.158	0.023	0.052; 0.683
Backward digit span				
Time × jitter^*^	−0.433	0.185	0.022	−0.800; −0.065
Time × shimmer^**^	0.213	0.079	0.008	0.056; 0.371

** Correlation is significant at to 0.0 level (2-tailed).

* Correlation is significant at to 0.05 level (2-tailed).

## 4 Discussion

The present study adds to the growing literature by suggesting that when using narrative speech, voice biomarkers are significantly related to longitudinal cognitive changes in a healthy community sample. Our study used narrative speech from a logical memory test, which was administered on average 8 years before cognitive outcomes and not included as a cognitive outcome measure. The results show that higher jitter and lower shimmer measured 8 years before cognitive outcomes predicted individual differences in 10-year cognitive changes in a national sample of community-residing middle-aged and older adults. The present study contributes to the expanding body of literature indicating that voice biomarkers, previously associated with dementia, are related to significant changes over approximately 10 years when assessed using narrative speech. Our findings were observed in a sample of healthy, community-dwelling middle-aged and older adults.

The jitter results in this study were consistent with prior research suggesting jitter gradually increases with age (Baken and Orlikoff, 2000). They also align with our earlier work, which found higher jitter assessed at the cognitive outcomes 10 years later was associated with greater 10-year cognitive declines (Mahon and Lachman, 2022). These jitter results were consistent across multiple tests of episodic memory and working memory. Though the shimmer findings were contrary to the expected direction, they were nonetheless consistently significant across multiple tests of episodic memory and working memory. The relationship between shimmer and cognitive decline has been relatively unexplored, particularly in a healthy community sample, making this an area of interest for further research. Typically, shimmer levels increase with age, even when controlling for health factors (Linville, 2004). However, the current results suggest that individuals with higher shimmer experience less cognitive decline compared to those with lower shimmer, when controlling for age and neurological conditions.

It is encouraging that by using prosody voice measures from narrative speech assessed approximately 8 years before cognitive outcomes, we were able to replicate and extend previous findings linking jitter with cognitive decline. These results suggest that it is feasible to predict cognitive decline through the analysis of voice metrics from speech samples collected with community-dwelling adults. Past studies have indicated that jitter and shimmer show age-related changes (Wilcox and Horii, 1980; Baken, 2005; Dehqan et al., 2013; Lortie et al., 2015). The current study adds that these 2 voice measures are also related to cognitive changes associated with aging. There are also some limitations of the study to consider. Given that our sample included only 3.8% non-white participants, future research involving more diverse samples is needed to provide more generalizable results. Also, cognitive diagnoses were not available in the sample, and having only 2 occasions is not ideal for examining differential trajectories of cognitive change.

Voice changes are among the earliest indicators of cognitive decline (Lin et al., 2020), and various prosody voice measures have been identified as risk factors for more rapid cognitive declines (Mahon and Lachman, 2022). While previous studies have explored these indicators to improve the identification of cognitive decline in healthy middle-aged and older adults, a universally accepted approach for using prosody voice measurements in the early diagnosis of preclinical stages of impairment before overt symptoms has yet to be established. Natural, cost-effective, and easily accessible voice measures from speech are a promising tool for detecting early cognitive decline and monitoring long-term progression to impairment, potentially serving as an additional comprehensive longitudinal biomarker. Thus, identifying voice biomarkers in midlife that predict later-life risk could facilitate earlier interventions by enabling the modification of disease risk factors (National Academies, 2017). As the prevalence of cognitive impairment grows, illuminating biomarkers capable of distinguishing significant patterns of cognitive changes from normal aging could prove invaluable in delaying or reversing the progression of the disease.

## Data Availability

All MIDUS survey and cognitive data are archived and made publicly available via the University of Michigan Inter-university Consortium of Political and Social Research (ICPSR) https://www.icpsr.umich.edu/web/ICPSR/series/203 or the MIDUS Portal https://midus.colectica.org. The voice data are not publicly available.
